# Dataset for recognition of snail trails and hot spot failures in monocrystalline Si solar panels

**DOI:** 10.1016/j.dib.2019.104441

**Published:** 2019-08-28

**Authors:** Estefanía Alfaro-Mejía, Humberto Loaiza-Correa, Edinson Franco-Mejía, Andrés David Restrepo-Girón, Sandra Esperanza Nope-Rodríguez

**Affiliations:** Escuela de Ingeniería Eléctrica y Electrónica (EIEE), Facultad de Ingeniería, Universidad del Valle, Colombia

**Keywords:** Photovoltaic array inspection, Monocrystalline Si panels, Snail trails, Hot spot defects, Thermographic images analysis, Unmanned aerial vehicles

## Abstract

This article presents a dataset for thermal characterization of photovoltaic systems to identify snail trails and hot spot failures. This dataset has 277 thermographic aerial images that were acquired by a Zenmuse XT IR camera (7–13 μm wavelength) from a DJI Matrice 100 ^1^drone (quadcopter). Additionally, our dataset includes the next environmental measurements: temperature, wind speed, and irradiance. The experimental set up consisted in a photovoltaic array of 4 serial monocrystalline Si panels (string) and an electronic equipment emulating a real load. The conditions for images acquisition were stablished in a flight protocol in which we defined altitude, attitude, and weather conditions.

**Table:** Specifications table

Subject area	*Computer Science*
More specific subject area	*Computer Vision and Pattern Recognition*
Type of data	*Tables, JPG files*
How data was acquired	*Photovoltaic panel ERDM Solar 85W*^2^ *IR camera Zenmuse XT, on board UAV Matrice 100*^3^ *Pyranometer SP110 Apogee*^4^ *Electronic Load B&K Precision 8514*^5^ *Weather station WS-2090*^6^
Data format	*Raw thermal images (336x256 pixels resolution), jpg format.* *Temperature in °C degrees* *Irradiance in W/m*^*2*^ *Wind speed in m/s*
Experimental factors	*Weather and operation conditions (irradiance, temperature, wind speed, altitude, attitude)* *Geographic location: 3° 22′30´´ N, 76° 32′04´´ W* *Time window: 10:00 to 14:00 local time.*
Experimental features	*Temperature (26–32 °C)* *Irradiance (500–*1000 W/m^2^*)* *Wind speed (3–*5 m/s*)* *IR images (7.5-13.5*μm*band)* *A drone with a mid-IR camera was used to inspect photovoltaic (PV) array of 4 serial monocrystalline Si panels (string) supplying an emulated load, in order to identify snail trails and hot spot failures by processing thermographic image sequences. The low height of the UAV stationary position allow inspection of individual cells, and complementary weather information is useful to established experimental conditions.*
Data source location	*Cali, Valle del Cauca, Colombia, South America*
Data accessibility	*https://data.mendeley.com/datasets/82vzccxb6y/2*
Related research article	[1] S. Gallardo-Saavedra, E. Franco-Mejia, L. Hernández-Callejo, Ó. Duque-Pérez, H. Loaiza-Correa, and E. Alfaro-Mejia, “Aerial thermographic inspection of photovoltaic plants: Analysis and selection of the equipment,” in *ISES Solar World Congress 2017 - IEA SHC International Conference on Solar Heating and Cooling for Buildings and Industry 2017, Proceedings*, 2017

2 http://www.erdm-solar.com/.

3 https://www.dji.com/matrice100.

4 https://www.apogeeinstruments.com/sp-110-ss-self-powered-pyranometer/.

5 http://www.bkprecision.com/products/dc-electronic-loads/8514-1200-w-programmable-dc-electronic-load.html.

6 https://www.ambientweather.com/amws2090ip.html

**Table:** 

**Value of the data** • The dataset of images can be used to classify hot spots, snail trails and sound cells in solar panels. • The dataset can be used for image processing to implement different techniques of filter and segmentation. • The resolution of images is high enough for cells segmentation on panels, allowing classification of different conditions of each cell. • The dataset can be used to thermally characterize solar cells inside panels regarding weather and flight conditions.

## 1 Data

**Fig. 1:**
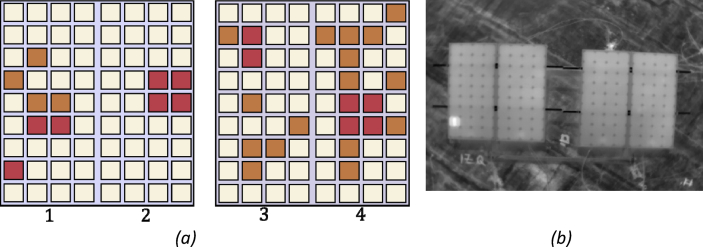
Four monocrystalline Si panels used for experiment: (a) scheme of panels with 9 × 4 cells in different conditions: hot spot (red) and snail trails (orange) failures, and sound cells (white), labeling of the panels, (b) Thermal image of the real solar panels.

The dataset is generated to thermal characterize snail trails and hot spot failures on solar panels of "monocrystalline Si" and is composed of 277 thermography images. The specifications of the equipment used in this research are presented in Table 1, Table 2, Table 3, Table 4, Table 5, Table 6. The information about the dataset is summarized in Table 7. This dataset is organized in folders named according to the acquisition date. Folders between April 28th to May 4th contain two subfolders: “Images” and “Irradiance_Hour”; the subfolder “Images” has only images from the left panel. However, the folder of May 4th contain a third subfolder named: “Temperature_WindSpeed”. Folders between December 20th to January 11th contain three subfolders: “Images”, “Irradiance_Hour” and “Temperature_WindSpeed”; the subfolder “Images” has the subfolders “Panels_right” and “Panels_left” containing images labeled as illustrated in Fig. 1; “Panels_right” correspond to panels (1,2), “Panels_left” correspond to panels (3,4) of the Fig. 1(a). All raw data are presented with the “.csv” extension. The “Irradiance_Hour” sub-folder contains a vector with information of the irradiance measurement, and the “Temperature_WindSpeed” sub-folder contains the wind speed measurement as well as the external and internal temperature.

**Table 1:** Solar panel specifications.

Number of cells	Dimensions (mm)	Weight (kg)	Open Circuit Voltage (V)	Short circuit current (A)
36	1186 × 551 × 35	9	21.78	5.13

**Table 2:** Electronic load specifications.

Resolution (mV/mA)	Minimum operating voltage (V)	Voltage Range (V)
1/0.1	0.1	0–120

**Table 3:** Pyranometer specifications.

Spectral range (nm)	Sensitivity ($mV\;W/m^{2}$)	Calibration Factor ($W/m^{2}\;mV$)	Field of view (degrees)	Operation Environment (°C)
360–1120	0.2	5	180	−40 a 70

**Table 4:** Weather station specifications.

Measurement	Range	Accuracy	Resolution
Outdoor Temperature	−10 to 149 °F (-23.3 to 65 °C)	± 2 °F ( ± 1.1 °C)	0.1 °F (0.06 °C)
Indoor Temperature	32 to 140 °F (0 to 60 °C)	± 2 °F ( ± 1.1 °C)	0.1 °F (0.06 °C)
Wind Direction	0-360°	22.5°	22.5°
Wind Speed	0 to 112 mph (0 to 180.3 km/h)	± 2.2 mph ( ± 3.5 km/h)	0.1 mph (0.16 km/h)

**Table 5:** Thermal camera specifications.

Pixels	Spectral Range (μm)	Angular vibration range	Thermal sensitivity (mK)	Weight (g)
336x256	7.5–13	± 0.03°	<50	270

**Table 6:** UAV specifications.

Type	Hovering time full payload (min)	Max speed of Ascent (m/s)	Max speed of Descent (m/s)	Operating Temperature (°C)
Quadcopter	20	5	4	−10 to 40

**Table 7:** Information about different groups of images conforming the Dataset.

Acquisition date	Number of images	Environmental variables	Altitude (m)	Attitude (°)	Reference file images
27-April-2018	13	Irradiance and Temperature.	2.3–2.7	45–60	27-April-2018
28-April-2018	13	Irradiance and Temperature	2.3–2.7	45–60	28-April-2018
04-May-2018	29	Irradiance and Temperature, wind speed	2.3–2.7	45–60	04-May-2018
20-December-2018	60	Irradiance and Temperature, wind speed	2.3–2.7	45–60	20-December-2018
21-December-2018	48	Irradiance and Temperature, wind speed	2.3–2.7	45–60	21-December-2018
16-January-2019	48	Irradiance and Temperature, wind speed	2.3–2.7	45–60	16-January-2019
19-January-2019	66	Irradiance and Temperature, wind speed	2.3–2.7	45–60	19-January-2019

**Fig. 2:**
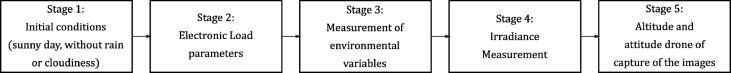
Images acquisition protocol.

**Fig. 3:**
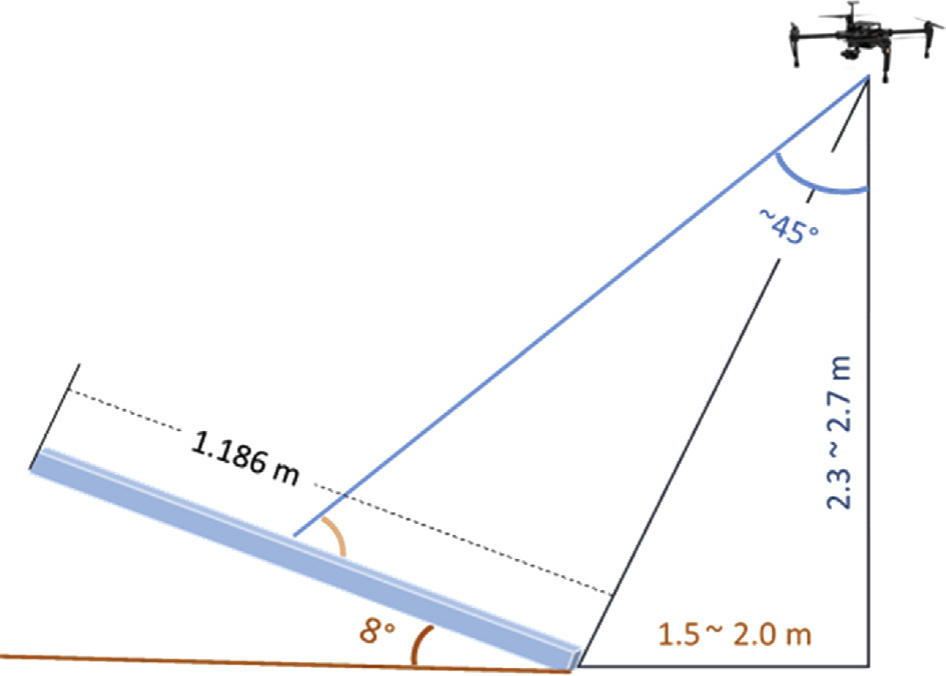
Positioning of UAV with respect to solar panels.

For its part, in Fig. 1(a) the conditions of the cells for the 4 panels inspected are highlighted by colors; in Fig. 1(b) a real thermal image of the panels is shown. In Fig. 2 the image acquisition protocol used is schematized. In Fig. 3 the relative position between UAV and solar panels is illustrated.

## 2 Experimental design, materials, and methods

### 2.1 Materials

For acquisition of the database the next materials and equipment were needed:

• String of 4 ERDM Si monocrystalline solar panel connected in series, with 85 W maximum power for each panel (Table 1).
• B&K Precision 8514 electronic load at 1200 W maximum power, to emulate a real load behavior (Table 2).
• SP110 Apogee pyranometer, placed besides the solar panels string, to measure irradiance (Table 3).
• Ambient Weather SW-2090 weather station, placed at 3 m height from solar panels surface, to measure temperature and wind speed (Table 4).
• Zenmuse XT thermal camera, to acquire thermographic images (Table 5).
• Matrice 100 drone, for aerial inspection of solar panels (Table 6).
• DJI Go application software, to visualize flight variables^7^

### 2.2 Method

An experimental protocol with 5 stages was designed to acquire the thermal images, as shown in Fig. 2.

#### 2.2.1 STAGE 1

According to [2], [3] the suggested irradiance level to acquire thermal images of photovoltaic systems (PV) should be at least 500$W/m^{2}$. Hence, images acquisition was performed in sunny days since clouds decrease the irradiance levels and rain is a flight restriction for UAV. Images were captured between 10:00 to 11:30 and 13:00 to 14:00 hours, in order to use the maximum peak of irradiance levels (∼ 800 W/m^2^) at Cali-Colombia. The interval between 11:30 to 13:00 hours was excluded to avoid shadows on the solar panels.

#### 2.2.2 STAGE 2

After checking the weather conditions, we set up the value of voltage at the B&K Precision 8514 electronic load (constant voltage operation mode) to 80% of the open-circuit voltage delivered by the solar panel string, in order to approximate the operation point of maximum power [4], [5].

#### 2.2.3 STAGE 3

It is necessary to verify that environmental variables are within suitable ranges, thus:

• Wind speed at 3–5 m/s, to guarantee the precision specification in temperature measurement calculated by the thermal camera sensor in this type of experiments [6].
• *Ambient temperature at a range of 26-30 °C, as suggested in* *[7]*.

#### 2.2.4 STAGE 4

At this stage, irradiance measurement is verified to be within the range: 500–1000 W/m^ˆ2^, as suggested in similar experiments [8].

#### 2.2.5 STAGE 5

Once environmental conditions are verified to be adequate for thermographic inspection, the UAV was positioned horizontally 2.0 m far apart the lowest side of panels, and at 2.3–2.7 m height from the base of the panels, as indicated by Fig. 3. Aiming to do the camera IFOV (1.889 mrad) to cover 2 times the area of a panel cell, an ideal height up to 2.3 m was established, similar as done in [9], although they did not consider the IFOV camera. In consequence, the thermograms resolution (336 × 256 pixels) is high enough to get information about cells condition, unlike other similar works [10], [11]where only global damages of panels can be detected because reported heights are greater than 20 m.

Additionally, inclination of the solar panels is 8°, which is possible due to geographical position of Cali, Colombia, close to equatorial line. This condition and a drone attitude (pitch angle) around 45° make the observation angle (between optical axis of camera and normal vector of the panel surface) be greater than 0° and smaller than 60° (45° approximately), in order to assure that emissivity variations are negligible [11].

Finally, because the drone is placed at a distance of up 2 m besides the panel, the cooling effect of the rotors blades of the UAV is negligible, and in consequence it is not taken into account.

### 2.3 Experimental design

We considered the images in dataset are spatially static because the drone was placed in fixed georeferenced coordinates, the pitch angle of the IR camera was constant, and height fluctuations with respect to the base of solar panels (2.3–2.7 m) were low, caused by gimbal vibrations or drone instability. However, acquisition was distributed in 7 experimental sessions, leading to important variations in irradiance, wind speed and ambient temperature, though all of them within allowable ranges. This situation aimed to recreate a similar uncontrolled environment than that found at outdoor solar panel installations. Table 7 specifies the values of flight and environmental variables for each group of thermal images captured during independent experimental sessions.

## Footnotes

7 https://www.dji.com/goapp.

## References

[1] Gallardo-Saavedra S., Franco-Mejia E., Hernández-Callejo L., Duque-Pérez Ó., Loaiza-Correa H., Alfaro-Mejia E. (2017). Aerial thermographic inspection of photovoltaic plants: analysis and selection of the equipment. ISES Solar World Congress 2017 - IEA SHC International Conference on Solar Heating and Cooling for Buildings and Industry 2017, Proceedings.

[2] Grimaccia F., Aghaei M., Mussetta M., Leva S., Quater P.B. (2015). Planning for PV plant performance monitoring by means of unmanned aerial systems (UAS). Int. J. Energy Environ. Eng..

[3] Quater P.B., Grimaccia F., Leva S., Mussetta M., Aghaei M. (2014). Light unmanned aerial vehicles (UAVs) for cooperative inspection of PV plants. IEEE J. Photovoltaics.

[4] Kontges M., Kurtz S., Ulrike J., Berger K.A., Kato K., Friesen T. (2015).

[5] Bastidas-Rodriguez J.D., Franco E., Petrone G., Ramos-Paja C.A., Spagnuolo G. (2017). Quantification of photovoltaic module degradation using model based indicators. Math. Comput. Simulat..

[6] Leva S., Aghaei M., Grimaccia F. (2015). PV Power Plant Inspection by UAS : Correlation between Altitude and Detection of Defects on PV Modules.

[7] Kauppinen T., Panouillot P.E., Siikanen S., Athanasakou E., Baltas P., Nikopoulous B. (2015).

[8] S. Dotenco et al., “Automatic Detection and Analysis of Photovoltaic Modules in Aerial Infrared Imagery.”.

[9] Álvarez-Tey G., Jiménez-Castañeda R., Carpio J. (2017). Analysis of the configuration and the location of thermographic equipment for the inspection in photovoltaic systems. Infrared Phys. Technol..

[10] Zhang P., Zhang L., Wu T., Zhang H., Sun X. (2017). Detection and location of fouling on photovoltaic panels using a drone-mounted infrared thermography system. J. Appl. Remote Sens..

[11] Buerhop C., Scheuerpflug H., Weissmann R. (2011). According to Fresnel’s law of glass optimal conditions for IR- imaging Of PV-plants. 26th Eur. Photovolt. Sol. Energy Conf. Exhib..

